# Limited predictability of maximal muscular pressure using the difference between peak airway pressure and positive end-expiratory pressure during proportional assist ventilation (PAV)

**DOI:** 10.1186/s13054-016-1554-4

**Published:** 2016-11-27

**Authors:** Po-Lan Su, Pei-Shan Kao, Wei-Chieh Lin, Pei-Fang Su, Chang-Wen Chen

**Affiliations:** 1 Section of Chest Medicine and Respiratory Care, Department of Internal Medicine, National Cheng Kung University Hospital, College of Medicine, National Cheng Kung University, Tainan, Taiwan; 2 Graduate Institute of Clinical Medical Sciences; Department of Respiratory Care, College of Medicine, Chang Gung University, Taoyuan, Taiwan; 3 Medical Intensive Care Unit, Department of Internal Medicine, National Cheng Kung University Hospital, College of Medicine, National Cheng Kung University, Tainan, Taiwan; 4 Department of Statistics, National Cheng Kung University, Tainan, Taiwan; 5 Medical Device Innovation Center, National Cheng Kung University, Tainan, Taiwan

**Keywords:** Pressure time product, Proportional assist ventilation, Airway pressure

## Abstract

**Background:**

If the proportional assist ventilation (PAV) level is known, muscular effort can be estimated from the difference between peak airway pressure and positive end-expiratory pressure (PEEP) (ΔP) during PAV. We conjectured that deducing muscle pressure from ΔP may be an interesting method to set PAV, and tested this hypothesis using the oesophageal pressure time product calculation.

**Methods:**

Eleven mechanically ventilated patients with oesophageal pressure monitoring under PAV were enrolled. Patients were randomly assigned to seven assist levels (20–80%, PAV20 means 20% PAV gain) for 15 min. Maximal muscular pressure calculated from oesophageal pressure (P_mus, oes_) and from ΔP (P_mus, aw_) and inspiratory pressure time product derived from oesophageal pressure (PTP_oes_) and from ΔP (PTP_aw_) were determined from the last minute of each level. P_mus, oes_ and PTP_oes_ with consideration of PEEPi were expressed as P_mus, oes, PEEPi_ and PTP_oes, PEEPi_, respectively. Pressure time product was expressed as per minute (PTP_oes_, PTP_oes, PEEPi_, PTP_aw_) and per breath (PTP_oes, br_, PTP_oes, PEEPi, br_, PTP_aw, br_).

**Results:**

PAV significantly reduced the breathing effort of patients with increasing PAV gain (PTP_oes_ 214.3 ± 80.0 at PAV20 vs. 83.7 ± 49.3 cmH_2_O•s/min at PAV80, PTP_oes, PEEPi_ 277.3 ± 96.4 at PAV20 vs. 121.4 ± 71.6 cmH_2_O•s/min at PAV80, *p* < 0.0001). P_mus, aw_ overestimates P_mus, oes_ for low-gain PAV and underestimates P_mus, oes_ for moderate-gain to high-gain PAV. An optimal P_mus, aw_ could be achieved in 91% of cases with PAV60. When the PAV gain was adjusted to P_mus, aw_ of 5–10 cmH_2_O, there was a 93% probability of PTP_oes_ <224 cmH_2_O•s/min and 88% probability of PTP_oes, PEEPi_ < 255 cmH_2_O•s/min.

**Conclusion:**

Deducing maximal muscular pressure from ΔP during PAV has limited accuracy. The extrapolated pressure time product from ΔP is usually less than the pressure time product calculated from oesophageal pressure tracing. However, when the PAV gain was adjusted to P_mus, aw_ of 5–10 cmH_2_O, there was a 90% probability of PTP_oes_ and PTP_oes, PEEPi_ within acceptable ranges. This information should be considered when applying ΔP to set PAV under various gains.

## Background

Although mechanical ventilation is a crucial tool in decreasing the respiratory effort required by ventilated patients, diaphragmatic weakness can rapidly develop with complete diaphragmatic inactivity and mechanical ventilation [1]. This type of diaphragmatic powerlessness has been termed ventilator-induced diaphragmatic dysfunction (VIDD) [2]. Controlled mechanical ventilation is a major factor in VIDD, which may be attenuated with assisted ventilation [3, 4]. This suggests that maintaining appropriate respiratory effort may be essential to preserving diaphragm function, and the ability to monitor respiratory effort during mechanical ventilation should be an important clinical issue [5].

Pressure applied to the respiratory system is usually assumed to dissipate against resistant and elastic elements. In a mechanically ventilated patient, the applied pressure is shared between the patient and ventilator [6]. This equation is difficult to solve under conventional ventilation because it is challenging to obtain reliable values for respiratory system resistance and elastance. However, in proportional assist ventilation (PAV), obtaining reliable elastance is possible during spontaneous breathing because the end of inspiration can be determined [7–9].

PAV with load-adjustable gain factors (PAV+) is a ventilatory mode that delivers assistance in proportion to the instantaneous flow and volume by calculating the instantaneous pressure needed to overcome the elastic and resistive pressures; these are updated several times per minute during PAV ventilation [10]. The proportion assistance is expressed as a percentage of the total pressure assisted (i.e. gain). By using this algorithm, Carteaux et al. [11] proposed a look-up table for estimating peak muscular pressure from peak airway pressure (P_aw, peak_) and positive end-expiratory pressure (PEEP) difference (ΔP), thus offering a way to keep the patient in a predefined comfort zone by adjusting the PAV gain. However, this algorithm has not yet been validated [12].

The oesophageal pressure time product (PTP_oes_) is a standard reference to assess respiratory muscle pressure. In patients with successful weaning, inspiratory PTP_oes_ is usually <224–255 cmH_2_O · s/min throughout the weaning trial [13]. In addition to possible variability in respiratory elastance and resistance measured during PAV+, respiratory muscular PTP as estimated by Carteaux’s method requires several assumptions that may limit its accuracy (e.g. a triangular muscular pressure waveform and a defined inspiratory time based on P_aw, peak_) [11]. Thus, the derived muscular PTP may not be equal to the PTP_oes_. The present study aimed to verify the applicability of Carteaux’s method with measured P_mus, oes_, P_mus, oes, PEEPi_, PTP_oes_, and PTP_oes, PEEPi_ under different PAV gain settings.

## Methods

From June 2014 to October 2014, all mechanically ventilated patients in our respiratory intensive care unit (10 beds) were screened daily for appropriateness for study inclusion. Patients had to be haemodynamically stable without inotropic agents and had to be ventilated with an inspiratory oxygen fraction <0.5 and PEEP ≤8 cmH_2_O. They also had to agree to oesophageal balloon placement. Exclusion criteria were pregnancy, acute coronary syndrome, aortic dissection as a cause of admission, and nasal or oropharyngeal lesions that prohibited oesophageal balloon placement. We used a single type of ventilator, the Puritan-Bennett 840 with PAV+ mode (Tyco International, Princeton, NJ, USA). The National Cheng Kung University Hospital Ethics Committee (A-BR-102-090) approved this study. The patient’s next of kin gave informed consent.

The oesophageal balloon was placed in the lower third of the oesophagus and inflated with 0.5–1 mL of air. Airflow was measured via a pneumotachograph (PN 155362, Hamilton Medical, Bonaduz, Switzerland), while the airway and oesophageal pressures were individually measured using two differential pressure transducers (P/N 113252, Model 1110A, Hans Rudolph, Shawnee, KS, USA). The flow sensor was placed between the endotracheal tube and ventilator Y-piece. Tidal volume was obtained by integration of the flow signal. All signals were sampled and digitalized at 100 Hz, and data were stored in a data-acquisition system (AcqKnowledgement, Biopac MP150, Goleta, CA, USA). All patients were assessed in a 30° supine position with endotracheal suction performed before measurement if clinically required.

For individual patients, seven PAV gain levels (percentage of assistance), namely PAV20 (20% gain), PAV30, PAV40, PAV50, PAV60, PAV70, and PAV80, were randomly applied for 15 min at each level unless the patients showed discomfort. Respiratory mechanics measured by the ventilator during PAV were recorded throughout the course. Passive respiratory mechanics were measured under constant flow at the end of this protocol by increasing the back-up mandatory ventilator rate until all the breathing efforts were suppressed [13, 14].

### Physiological measurement

#### Validation of oesophageal pressure measurement

Appropriate oesophageal balloon placement was verified by the occlusion test [15]. The ratios of change in oesophageal pressure to the change in airway opening pressure (ΔP_oes_/ΔP_aw_) during three to five spontaneous respiratory efforts against a closed airway were determined to ensure oesophageal pressure measurement reliability.

#### Respiratory mechanics during PAV and passive mechanical ventilation

The respiratory mechanics (E_pav_ and R_pav_) during different PAV levels were recorded as a display on the ventilator screen. The last five E_pav_ and five R_pav_ at each PAV level were used for comparison. The respiratory system mechanics under constant flow and volume-cycled passive mechanical ventilation were determined at the end of the protocol using constant flow and a rapid airway occlusion technique [16, 17].

#### Maximum inspiratory muscular pressure with P_oes_ tracing (P_mus, oes_) and inspiratory oesophageal pressure time product per breath (PTP_oes, br_)

**Fig. 1:**
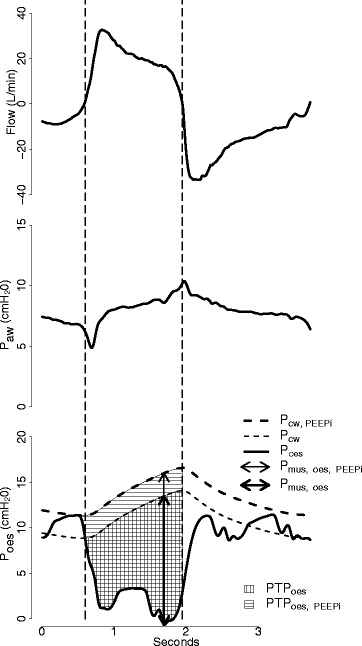
Graphic illustration of flow, airway pressure (*P*_*aw*_), and oesophageal pressure tracing (*P*_*oes*_) during proportional assist ventilation. Chest wall recoil pressure (*P*_*cw*_) was calculated from the product of tidal volume and dynamic chest wall elastance. Upper bound oesophageal pressure time product (*PTP*_*oes*, *PEEP*i_) was calculated as the integration of the difference between P_cw, PEEPi_ and P_oes_. Lower bound oesophageal pressure time product (*PTP*_*oes*_) was calculated as the integration of the difference between P_cw_ and P_oes_. P_mus, oes_ and P_mus, oes, PEEPi_ represent the maximal difference between passive and active P_oes_

Muscular pressure was calculated by taking into account dynamic E_cw_, which was obtained as the passive volume-oesophageal pressure slope [13]. P_mus, oes_ was defined as the maximum difference between the passive and active P_oes_. The inspiratory PTP_oes_ was calculated as the area between the P_cw_ and P_oes_ tracing, starting from the onset of inspiratory effort to the end of inspiratory flow. P_cw_ was obtained by multiplying the tidal volume by dynamic E_cw_. The onset of inspiratory effort was determined by the rapid descent point from P_oes_. We calculated PTP_oes_ with and without consideration of the intrinsic PEEP (PEEPi) [13]. Because gastric pressure was not measured, exact amounts of dynamic hyperinflation and expiratory muscle activity were unknown. The PTP_oes_ was thus presented in two forms, the upper bound PTP_oes_, which attributes the rapid descent of P_oes_ before the onset of inspiratory flow solely to inspiratory muscle activity, and the lower bound PTP_oes_, which attributes the rapid descent of P_oes_ solely to cessation of expiratory effort [13, 14]. PTP_oes, PEEPi_ and PTP_oes_ thus represent the upper and lower bounds of PTP, respectively (Fig. 1).

#### Maximum inspiratory pressure from Δ*P and PAV gain (P*_*mus, aw*_*) and inspiratory pressure time product from airway per breath (PTP*_*aw, br*_*)*

P_mus, aw_ during PAV was obtained by using the formula adopted by Carteaux [11]:

$$ {\mathrm{P}}_{\mathrm{mus},\ \mathrm{aw}} = \left({\mathrm{P}}_{\mathrm{aw},\ \mathrm{peak}} - \mathrm{PEEP}\right) \times \left(100 - \mathrm{gain}\right)/\mathrm{gain}. $$

PTP_aw, br_ was calculated under the assumption of a triangular inspiratory path with the end of inspiratory effort at P_aw, peak_.

### Statistical analysis

The results are given as mean ± SD, unless otherwise specified. The Kruskal-Wallis test was used to compare means from different groups. Dunn’s multiple comparison test was performed over pairs of groups. Repeated measured analysis of variance (ANOVA) was used to compare the means of E_pav_ and R_pav_ measured by the ventilator during various PAV gain levels. Correlatios between PTP_oes, br_ and P_mus, oes_, PTP_oes, PEEPi, br_ and P_mus, oes, PEEPi_, and PTP_aw, br_ and P_mus, aw_ were analysed using the two-tailed Spearman correlation test. Linear regression between PTP_oes, br_ and P_mus, oes_, PTP_oes, PEEP, br_ and P_mus, oes, PEEPi_, and PTP_aw, br_ and P_mus, aw_ was analysed with a forced regression line through the origin. Limits of agreement between P_mus, aw_ and P_mus, oes_ were examined using Bland-Altman analysis. All tests were two-sided, and a *p* value less than .05 was considered statistically significant. All analyses were performed using Prism version 5 (GraphPad Software, San Diego, CA, USA).

## Results

**Fig. 2:**
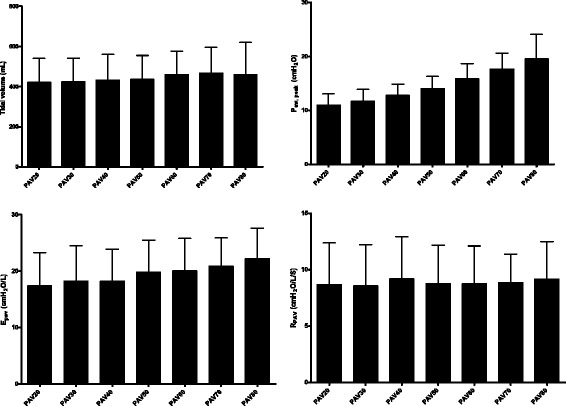
Tidal volume, peak airway pressure (*P*_*aw*, *peak*_) and respiratory mechanics during proportional assist ventilation (*PAV*) under different gains. *PAV20* indicates a mean gain level of 20%. Significant differences in tidal volume were found between PAV60 vs. PAV20, PAV70 vs. PAV20, PAV70 vs. PAV30, and PAV70 vs. PAV40. Significant differences in P_aw, peak_ were found among individual P_aw, peak_ levels under different gains, except the P_aw, peak_ of PAV20 vs. P_aw, peak_ of PAV30 and P_aw, peak_ of PAV70 vs. P_aw, peak_ of PAV80. For PAV-based patient elastance (*E*_*pav*_), significant differences were found between PAV20 vs. PAV50, PAV60, PAV70, and PAV80; PAV30 vs. PAV50, PAV60, PAV70, and PAV80; PAV40 vs. PAV50, PAV60, PAV70, and PAV80; PAV50 vs. PAV70 and PAV80; PAV60 vs. PAV80; and PAV70 vs. PAV80. No significant difference was found in PAV-based patient resistance (*R*_*pav*_) among various gains. For the E_pav_ and R_pav_ comparison, one patient was not included because of insufficient numbers of E_pav_ and R_pav_ in PAV20 and PAV30

The results of 18 consecutive patients who fulfilled the inclusion criteria were recorded. Two patients were excluded from further analysis because of a low ΔP_oes_/ΔP_aw_ ratio. One patient was excluded because of a poor oesophageal pressure signal, and four patients were excluded because of an inadequate duration of P_oes_ tracing secondary to the intolerance of the patients to low-gain PAV. Ultimately, 11 patients with an adequate duration of PAV recording at all stages of PAV support were analysed. The clinical demographics and respiratory mechanics of these patients are shown in Table 1. The tidal volume, P_aw, peak_, E_pav_, and R_pav_ under various PAV gain levels are shown in Fig. 2. Significantly higher tidal volumes were found with high PAV gains. As predicted, P_peak_ increased with PAV gain. There were no significant changes in R_pav_, but E_pav_ was significantly higher with a high PAV gain (*p* < 0.0001).

**Table 1:** Patient demographics and respiratory mechanics

Case	Age (years)/Sex	Diagnosis	Days on MV/ETT size (mm)/ΔP_ETT_ (cmH_2_O)	Baseline FiO_2_/PEEP (cmH_2_O)	E_rs_ (cmH_2_O/L)	E_cw_ (cmH_2_O/L)	R_max_ (cmH_2_O/L/S)	R_min_ (cmH_2_O/L/S)	ΔP_oes_/ΔP_aw_
1	61/M	Emphysema, dementia	13/7.5/3.73	0.35/0	21.71	9.96	22.38	10.37	0.94
2	88/M	Pneumonia, COPD	7/7.0/4.92	0.40/8	15.28	5.57	27.11	21.55	1.03
3	80/F	UTI	9/7.5/3.61	0.25/8	18.24	3.93	10.05	7.99	1.00
4	67/F	MRSA bacteraemia	8/7.5/4.05	0.30/6	32.62	9.52	11.08	7.21	1.11
5	80/F	UTI, old stroke	4/7.5/5.36	0.40/6	21.18	6.06	22.29	17.93	0.91
6^b^	88/F	UTI, CHF	4/7.5/5.99	0.40/6	30.51	12.54	21.26	16.73	0.92
7	54/F	Pneumonia, old stroke	18/7.0^a^/1.97^a^	0.30/6	23.04	5.13	17.25	13.69	1.03
8	67/M	Pneumonia	4/7.5/3.93	0.40/6	16.74	3.00	12.46	9.31	0.92
9	79/F	Pneumonia, CHF	8/7.5/6.07	0.30/6	23.98	6.28	22.02	16.95	0.81
10	74/M	COPD	3/7.5/3.71	0.30/6	7.73	4.24	23.52	13.35	1.11
11^c^	84/F	UTI, parkinsonism, asthma	4/7.5/3.86	0.35/6	21.61	6.72	20.88	13.34	0.77

^a^Tracheostomy tube and ΔP_ETT_ only an approximation as equation only available for an 8.0-mm tracheostomy. ^b^Cheyne-Stokes breathing noted. ^c^Evident abdominal muscle contraction noted. *FiO*_*2*_ inspired oxygen fraction, *ETT* endotracheal tube, *CHF* congestive heart failure, *COPD* chronic obstructive pulmonary disease, *E*_*rs*_ passive respiratory system elastance, *E*_*cw*_ passive chest wall elastance, *F* female; *M* male, *MV* mechanical ventilation, *MRSA* methicillin-resistant *Staphylococcus aureus*, *PEEP* positive end-expiratory pressure, *Rmax* passive maximum end-inspiratory resistance, *Rmin* passive minimum (airway) end-inspiratory resistance, *UTI* urinary tract infection, *ΔP*_*oes*_ */ΔP*_*aw*_ ratio of oesophageal pressure drop to airway pressure drop during airway occlusion, *ΔP*_*ETT*_ pressure loss through endotracheal or tracheostomy tube

### *PTP*_*oes*_*, PTP*_*oes, PEEPi*_*, peak muscular pressure and duration of inspiration (Ti) with different PAV gains and their correlation analysis*

**Fig. 3:**
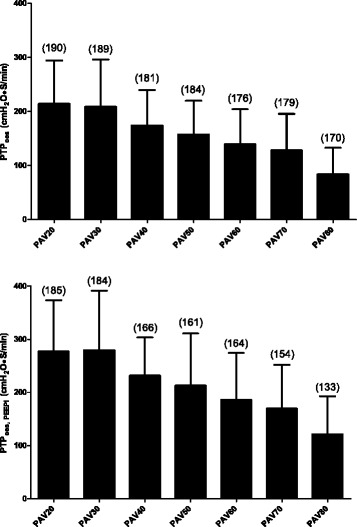
Inspiratory pressure time product (*PTP*) under different gain levels. PTP calculated from the difference between the oesophageal pressure and the relaxed chest wall elastance curve (*PTP*_*oes*_) decreased progressively with increasing gain with or without intrinsic positive end-expiratory pressure (*PEEPi*). For PTP_oes_, a significant difference was found between proportional assist ventilation 20% gain (*PAV20*) vs. PAV40, PAV50, PAV60, PAV70, and PAV80; PAV30 vs. PAV50, PAV60, PAV70, and PAV80; PAV40 vs. PAV60, PAV70, and PAV80; PAV50 vs. PAV70 and PAV80; PAV60 vs. PAV80; and PAV70 vs. PAV80. Similar patterns were found with PTP_oes, PEEPi_. Values in *parentheses* are the number of breaths analysed in each gain level

**Fig. 4:**
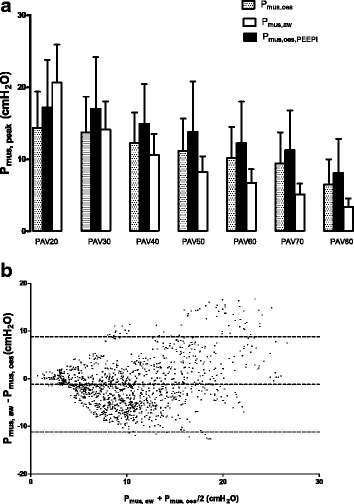
**a** Maximum muscular pressure (*P*_*mus*_) determined using either oesophageal pressure tracing or airway pressure under different proportional assist ventilation (PAV) gains. Significant differences (*p* < 0.05) were observed for all gain levels. **b** Bland-Altman analysis plot showing bias and agreement between maximal muscular pressure calculated from ΔP and PAV gain (*P*_*mus, aw*_) and maximal muscular pressure calculated from maximum difference between passive and active P_oes_ without consideration of PEEPi (*P*_*mus*, *oes*_). The *middle dashed line* is the mean difference (bias). The *outer dashed line* is the 95% confidence interval of the difference between P_mus, aw_ and P_mus, oes_ (±1.96 SD)

PTP_oes_ and PTP_oes, PEEPi_ during various PAV gain factors are shown in Fig. 3. Progressive reductions in PTP_oes_ and PTP_oes, PEEPi_ were noted with increasing PAV gain levels. Significant differences were found among those with low-gain and high-gain PAV (*p* < 0.0001). However, no significant difference in PTP_oes_ or PTP_oes, PEEPi_ was found between PAV20 vs. PAV30, PAV30 vs. PAV40, PAV40 vs. PAV50, or PAV50 vs. PAV60. P_mus, aw_ tended to underestimate P_mus, oes_ or P_mus, oes, PEEPi_ with all levels of PAV gain except PAV20 (Fig. 4a). The minimal difference between P_mus, aw_ and P_mus, oes_ was at the level of PAV30 (Fig. 4a). The T_i, aw_ estimated from the onset of inspiratory effort to P_aw, peak_ was not different from that estimated from flow tracing from PAV20 to PAV50. However, the T_i, aw_ was significantly shortened compared to the Ti estimated from flow tracing within PAV60 to PAV80 (data not shown, *p* < 0.0001). Spearman correlation analysis revealed significant correlation between P_mus, aw_ and PTP_aw, br_ (*r* ^2^ = 0.9341), P_mus, oes_ and PTP_oes, br_(*r* ^2^ = 0.8751), and P_mus, oes, PEEPi_ and PTP_oes, PEEPi, br_ (*r* ^2^ = 0.8862). Linear regression analysis disclosed the best-fit slope between PTP_aw, br_ and P_mus, aw_ to be 0.56, between PTP_oes, br_ and P_mus, oes_ to be 0.73, and between PTP_oes,_ _PEEPi,_ _br_ and P_mus, oes, PEEPi_ to be 0.83.

#### Bland-Altman analysis of P_mus_ between P_mus, aw_ and P_mus, oes_ and selection of optimal P_mus_

There was limited agreement between P_mus, aw_ and P_mus, oes_ as determined by Bland-Altman analysis (Fig. 4b). The bias was -1.2 cmH_2_O. The 95% confidence interval between P_mus, aw_ and P_mus, oes_ was from -11.2 to 8.8 cmH_2_O. The maximal muscular pressures estimated from three different approaches under different PAV gain levels are shown in Table 2. PAV60 was associated with the highest probability (91%) of optimal P_mus_ according to P_mus, aw_ (5–10 cmH_2_O). However, the best PAV gain for optimal PAV assessed from P_mus, oes_ or P_mus, oes, PEEPi_ was quite diverse and was absent in two patients. The concordance rate for selection of optimal PAV gain was <50% between P_mus, aw_ and P_mus, oes_ and P_mus, aw_ and P_mus, oes, PEEPi_.

**Table 2:** Maximal muscular pressures determined through airway or oesophageal pressure with and without PEEPi

Case	P_mus_ and PEEPi (cmH_2_O)	PAV20	PAV30	PAV40	PAV50	PAV60	PAV70	PAV80
1	P_mus, aw_	25	18	15	11	9	7	4
	P_mus, oes_	20	19	18	13	15	13	7
	P_mus, oes, PEEPi_	21	22	19	14	16	14	8
	PEEPi level	1.5 ± 0.8	2.6 ± 0.8	1.4 ± 0.5	0.7 ± 0.5	1.1 ± 0.7	1.4 ± 0.8	0.3 ± 0.3
2	P_mus, aw_	19	13	10	8	6	5	3
	P_mus, oes_	16	15	16	13	13	14	11
	P_mus, oes, PEEPi_	17	17	17	14	15	15	12
	PEEPi level	1.6 ± 0.7	1.1 ± 0.6	1.0 ± 0.6	1.4 ± 0.6	1.3 ± 0.8	1.3 ± 0.6	1.8 ± 0.6
3	P_mus, aw_	17	10	8	6	5	4	3
	P_mus, oes_	17	14	13	14	11	11	8
	P_mus, oes, PEEPi_	18	16	14	15	12	14	9
	PEEPi level	1.6 ± 0.9	1.9 ± 1.0	1.0 ± 0.8	0.7 ± 0.8	1.0 ± 0.8	1.4 ± 1.1	0.3 ± 0.6
4	P_mus, aw_	17	13	10	8	6	5	3
	P_mus, oes_	14	16	11	11	10	8	5
	P_mus, oes, PEEPi_	15	17	13	12	11	9	6
	PEEPi level	1.3 ± 0.9	1.2 ± 1.0	1.3 ± 0.9	1.7 ± 1.2	0.8 ± 0.4	1.2 ± 1.3	0.6 ± 0.5
5	P_mus, aw_	13	9	7	5	5	4	3
	P_mus, oes_	9	9	8	7	8	6	4
	P_mus, oes, PEEPi_	10	10	9	8	8	6	4
	PEEPi level	0.3 ± 0.2	0.4 ± 0.4	0.0 ± 0.1	0.1 ± 0.1	0.3 ± 0.4	0.0 ± 0.1	0.1 ± 0.1
6	P_mus, aw_	23	18	13	11	9	7	6
	P_mus, oes_	21	21	17	20	17	14	13
	P_mus, oes, PEEPi_	27	28	19	27	23	18	18
	PEEPi level	6.3 ± 4.0	7.4 ± 3.9	2.7 ± 1.4	6.8 ± 4.5	6.3 ± 4.8	4.2 ± 2.8	4.4 ± 3.2
7	P_mus, aw_	14	9	7	5	4	3	2
	P_mus, oes_	4	5	4	4	3	2	2
	P_mus, oes, PEEPi_	7	7	7	6	4	4	3
	PEEPi level	3.0 ± 0.9	2.9 ± 0.8	3.0 ± 1.5	2.1 ± 0.7	1.6 ± 0.8	1.5 ± 0.5	1.3 ± 0.8
8	P_mus, aw_	20	14	10	8	6	5	3
	P_mus, oes_	14	13	10	10	8	7	7
	P_mus, oes, PEEPi_	16	15	12	10	9	8	8
	PEEPi level	2.8 ± 0.7	2.2 ± 0.6	1.9 ± 0.8	0.8 ± 0.5	0.9 ± 0.3	1.0 ± 0.4	0.8 ± 0.4
9	P_mus, aw_	26	18	13	9	8	5	3
	P_mus, oes_	12	11	10	8	8	6	4
	P_mus, oes, PEEPi_	14	13	12	10	10	8	6
	PEEPi level	2.1 ± 0.7	2.0 ± 0.7	2.0 ± 0.7	1.8 ± 0.6	2.1 ± 0.5	1.6 ± 0.5	1.7 ± 0.4
10	P_mus, aw_	26	14	9	8	6	5	4
	P_mus, oes_	16	15	15	13	11	11	9
	P_mus, oes, PEEPi_	17	16	16	14	12	12	11
	PEEPi level	1.2 ± 1.0	1.2 ± 1.1	1.0 ± 1.0	0.4 ± 0.6	0.8 ± 0.8	0.1 ± 0.3	0.2 ± 0.5
11	P_mus, aw_	28	19	14	12	10	7	4
	P_mus, oes_	16	14	13	12	8	12	5
	P_mus, oes, PEEPi_	28	32	29	28	17	18	10
	PEEPi level	12.0 ± 1.3	17.9 ± 3.5	16.2 ± 1.7	15.9 ± 2.9	8.6 ± 1.7	6.0 ± 1.9	5.0 ± 1.4

Maximum muscular pressure and intrinsic positive end-expiratory pressure (PEEPi) were calculated as average of 1-minute breaths in each proportional assist ventilation (PAV) gain. Muscular pressures between 5 and 10 cmH_2_O are highlighted. *P*_*mus*, *aw*_ maximal muscular pressure calculated from ΔP and PAV gain, *P*_*mus, oes*_ maximal muscular pressure calculated from maximum difference between passive and active P_oes_ without consideration of PEEPi, *P*_*mus, oes, PEEPi*_ maximal muscular pressure calculated from maximum difference between passive and active P_oes_ with consideration of PEEPi

P_mus,aw_ within 5–10 cmH_2_O was not present in PAV20 but was present in 11–82% of breaths in other PAV gains. Around 80% of breaths in PAV50 or PAV60 were associated with P_mus,aw_ within 5–10 cmH_2_O. PTP_oes_ <224 cmH_2_O·s/min and PTP_oes, PEEPi_ <255 cmH_2_O·s/min are considered admissible according to Jubran et al. [13]. Despite the limited predictability of P_mus, oes_ or P_mus, oes, PEEPi_ from P_mus, aw_, patients with P_mus, aw_ between 5 and 10 cmH_2_O are had 93% probability of PTP_oes_ <220 cmH_2_O·s/min and 88% probability of PTP_oes, PEEPi_ <255 cmH_2_O·s/min, regardless of the PAV gain. Only two breaths were associated with PTP_oes_ values <40 cmH_2_O·s/min. When P_mus,aw_ was achieved within 5–10 cmH_2_O, three PAV gain levels (PAV40, PAV50 and PAV60) were associated with >90% probability of admissible PTP_oes_ and PTP_oes, PEEPi_.

## Discussion

Our analyses revealed several interesting findings. First, PTP_oes_ and PTP_oes, PEEPi_ significantly decreased with increasing PAV gain in patients with PAV. Second, the prediction of P_mus, oes_ or P_mus, oes, PEEPi_ from airway pressure tracing had limited accuracy. Third, the deduction of PTP_aw_ from ΔP may underestimate PTP_oes_ or PTP_oes, PEEPi_. Fourth, an optimal P_mus, aw_ (5–10 cmH_2_O) could be achieved in 91% of patients with PAV60, and despite the lack of accuracy for predicting P_mus, oes_ or P_mus, oes, PEEPi_ from airway pressure tracing, maintaining P_mus, aw_ within 5–10 cmH_2_O was associated with PTP_oes_ <224 cmH_2_O·s/min or PTP_oes, PEEPi_ <255 cmH_2_O·s/min in approximately 90% of breaths.

The significant increase in P_aw, peak_ but minimal difference in tidal volume with increasing gain level indicates substantial adaptation of muscular pressure during PAV [18]. The lower elastance during low assist could be explained by high respiratory drive (i.e. inspiratory muscle activity does not return to zero during the 300 ms occlusion time), which underestimates the elastic recoil pressure at end-inspiration. PEEPi is unlikely to be a cause because it did not increase with greater PAV assist in the current study [9].

The algorithm proposed by Carteaux et al. [11] is a simple bedside approach to estimate inspiratory muscular pressure (P_mus, aw_) in mechanically ventilated patients under PAV. We found it to be of limited value in predicting P_mus, oes_. P_mus, aw_ tends to overestimate P_mus, oes_ in PAV20 but more commonly underestimates P_mus, oes_ from PAV40 to PAV80. Therefore, the proportion of alleviation of respiratory muscle output was usually incompletely attained as the PAV gain intended it to be. Besides, the wide 95% confidence interval from the Bland-Altman analysis of P_mus, oes_ and P_mus, aw_ implicated that P_mus, oes_ could not be accurately predicted by P_mus, aw_.

There are several possible explanations for these findings. First, for the unique condition where P_mus, oes_ is usually overestimated in PAV20, a reasonable cause could be the ventilator flow control algorithm. Because respiratory effort is maximal in PAV20, the proportional-integral-derivative algorithm of the flow control system is prone to an airway pressure overshoot by the end of inspiration, which is further exaggerated fourfold in PAV20 for the calculation of P_mus, aw_ [19, 20]. Second is a possible discrepancy between PAV+ and CMV measured respiratory mechanics [10]. Although the PAV+ mode was continuously updated, measured respiratory system resistance and elastance may be different from those obtained under CMV [10]. Moreover, the respiratory system resistance measured by PAV+ is not reliable in cases with severe expiratory flow limitations. Third is the presence of PEEPi. In a recently published PAV+ mode bench study [21], the assistance provided by PAV+ was approximately 25% lower than expected. PEEPi with the associated trigger delay was considered a major factor affecting PAV+ accuracy due to the lack of assist during the initial part of respiratory breath, ultimately resulting in global under-assistance.

PTP_oes_ is a better surrogate of respiratory effort in ventilated patients. In this study, the analyses of correlation between P_mus, aw_ and PTP_aw_, P_mus, oes_ and PTP_oes_, P_mus, oes, PEEPi_ and PTP_oes, PEEPi_ yielded highly significant results. However, predicting PTP from P_mus, aw_ and P_mus, oes_ differed in the best-fit slope value. The slope value was 0.56 when the linear regression was performed between P_mus, aw_ and PTP_aw_. The slope increased to 0.73 between PTP_oes_, _br_ and P_mus, oes_ and to 0.83 between PTP_oes_, _PEEPi_, _br_ and P_mus, oes, PEEPi_. This implicates that the PTP_aw_ should be corrected when projecting into PTP_oes_. We offer the following explanation for the discrepancy between PTP_aw_ and PTP_oes_. First, the assumption of a triangular pressure-time product is flawed because respiratory muscle pressure generation is usually exponential [22–24]. The integration area above an exponential decay curve is usually larger than the integration area above a triangular line. Second, the inspiratory time is significantly shortened in high-gain PAV. The shortened inspiratory time should result in a smaller PTP_aw_ from the triangular algorithm. A third possible cause is the influence of PEEPi. The algorithm proposed by Cardeaux et al. is also flawed as it does not consider PEEPi. The inclusion of PEEPi led to increases in P_mus, oes, PEEPi_ and PTP_oes, PEEPi_.

The predefined range of respiratory effort by Carteaux and colleagues [11] needs critical appraisal. Target limits of P_mus, aw_ within 5–10 cmH_2_O or PTP_aw_ between 50 and 150 cmH_2_O·s/min were derived mainly from a desirable inspiratory effort of PTP_oes, PEEPi_ <125 cmH_2_O·s/min [14]. This recommended threshold is arbitrary, not supported by quantitative diaphragm electromyogram, and possibly well below the threshold of threatening diaphragm fatigue [14]. A wider range of PTP_oes, PEEPi_ should be allowable with minimal risk of diaphragm fatigue [13, 25, 26]. As P_mus, aw_ frequently underestimates P_mus, oes_ in the usual levels of PAV, actual PTP_oes, PEEPi_ values are usually higher than PTP_aw_. Interestingly, PTP_oes, PEEPi_ measurements were usually <255 cmH_2_O·s/min when P_mus, aw_ were within 5–10 cmH_2_O. This implicates that the recommended grid table for PAV remains a helpful reference for selecting the PAV level, although the newly advocated threshold requires further study for verification.

There are several limitations to the current study. The first is the limited number of patients studied and the fact that all of the patients had started to have weaning trials as reflected by the oxygen fraction and external PEEP level. Thus, our results may not be applicable to acutely ill patients under mechanical ventilation. The second is the lack of gastric pressure measurement, which meant that we could not clarify the contribution of expiratory muscle activity during PAV. However, we did not notice evident abdominal muscle contraction during PAV except in one patient with high PEEPi. Thus, the measured P_mus, oes, PEEPi_ should represent the inspiratory muscle motor outputs for most of our patients.

## Conclusions

In summary, our results demonstrate limited accuracy of estimating respiratory effort from airway pressure tracing during PAV. Although P_mus, oes_ decreases with increasing PAV gain, P_mus, oes_ could not be precisely predicted from ΔP under various gain factors. In addition, PTP_aw_ also underestimated PTP_oes_ and PTP_oes, PEEPi_. However, when the PAV gain was adjusted to a P_mus, aw_ of 5–10 cmH_2_O, there was approximately 90% probability of maintaining the patient within an acceptable PTP range.

## Abbreviations

CMV: continuous mandatory ventilation
E_cw_: passive chest wall elastance during CMV
E_pav_: PAV-based patient elastance
E_rs_: passive respiratory system elastance during CMV
PAV: proportional assist ventilation
PAV20 to PAV80: 20 to 80% PAV gain
P_aw, peak_: peak airway pressure during PAV
P_cw_: chest wall elastic pressure
PEEP: positive end-expiratory pressure
PEEPi: intrinsic PEEP
P_mus, aw_: maximal muscular pressure calculated from ΔP and PAV gain
P_mus, oes_: maximal muscular pressure calculated from maximum difference between passive and active P_oes_ without consideration of PEEPi
P_mus, oes, PEEPi_: maximal muscular pressure calculated from maximum difference between passive and active P_oes_ with consideration of PEEPi
P_mus_: respiratory muscular pressure
PTP: inspiratory pressure time product
PTP_aw, br_: inspiratory pressure time product calculated from ΔP and assuming a triangular inspiratory pressure time course per breath
PTP_aw_: inspiratory pressure time product calculated from ΔP and assuming a triangular inspiratory pressure time course
PTP_oes, br_: inspiratory pressure time product calculated from the difference between the oesophageal pressure and the relaxed chest wall elastance curve per breath
PTP_oes_: inspiratory pressure time product calculated from the difference between the oesophageal pressure and the relaxed chest wall elastance curve
PTP_oes, PEEPi, br_: inspiratory pressure time product calculated from the difference between the oesophageal pressure and the relaxed chest wall elastance curve per breath with consideration of PEEPi
PTP_oes, PEEPi_: PTP_oes_ with consideration of PEEPi
R_max_: passive maximum inspiratory resistance during CMV
R_min_: passive minimum (airway) inspiratory resistance during CMV
R_pav_: PAV-based patient resistance
T_i_: duration of the inspiratory time determined from flow tracing during various PAV gains without consideration of PEEPi
T_i, aw_: duration of the inspiratory time determined from the peak airway pressure during various PAV gains
VIDD: ventilator-induced diaphragmatic dysfunction
ΔP: peak airway pressure and PEEP difference
